# Single Infrared Image-Based Stripe Nonuniformity Correction via a Two-Stage Filtering Method

**DOI:** 10.3390/s18124299

**Published:** 2018-12-06

**Authors:** Qingjie Zeng, Hanlin Qin, Xiang Yan, Shuowen Yang, Tingwu Yang

**Affiliations:** School of Physics and Optoelectronic Engineering, Xidian University, Xi’an 710071, China; qingjie_zeng@126.com (Q.Z.); xyan@xidian.edu.cn (X.Y.); shuowen_yang@163.com (S.Y.); ytingwu@cfte.com.cn (T.Y.)

**Keywords:** infrared imaging, stripe nonuniformity, spectral analysis, filtering

## Abstract

The presence of stripe nonuniformity severely degrades the image quality and affects the performance in many infrared (IR) sensing applications. Prior works correct the nonuniformity by using similar spatial representations, which inevitably damage some detailed structures of the image. In this paper, we instead take advantage of spectral prior of stripe noise to solve its correction problem in single IR image. We first analyse the significant spectral difference between stripes and image structures and utilize this knowledge to characterize stripe nonuniformity. Then a two-stage filtering strategy is adopted combining spectral and spatial filtering. The proposed method enables stripe nonuniformity to be eliminated from coarse to fine, thus preserving image details well. Extensive experiments on simulated images and raw IR images demonstrate that the proposed method achieves superior correction performance over the recent state-of-the-art methods.

## 1. Introduction

In many infrared (IR) sensing applications such as target detection [1] and face recognition [2], one has to process images and videos containing undesirable fixed pattern noise (FPN) that is caused by the nonuniformity of IR detector response [3,4]. Hence, it is important for IR sensors to correct the nonuniformity and remove the FPN in raw images. In this paper, we address the problem of stripe nonuniformity correction within a single IR image.

Nonuniformity correction (NUC) has drawn increasing attention from the research community, owing to its good capability of eliminating the FPN. Many researchers have devoted their attention to developing various NUC methods over the past several decades. They have been roughly divided into two categories: the calibration based methods [5,6,7] and the scene based methods [8,9,10,11,12]. The former enables detectors to have a consistent response at the same temperature through the radiance calibration; the latter reduces the FPN via multi-frame images association. With the growing use of uncooled IR detectors, stripe nonuniformity has acted as a main FPN in IR images [4,13]. Many researchers are devoted to developing targeted methods to correct the nonuniformity within a single image. The mainstream methods focus on utilizing column directionality of stripes in spatial domain, such as midway infrared equalization (MIRE) [13], 1-D guided filtering [14] and total variation model [15]. Despite varying degrees of success, the majority of these methods suffer from several limitations:

(a) Due to the intrinsic overlapping between stripe nonuniformity and structural patterns in the image, most methods reduce the striping artifact but damage texture details more or less, leading to the over-smoothing regions.

(b) The basic operation of many existing algorithms is repeatedly estimating the nonuniformity on local spatial regions, which has not been proven to have high efficiency.

Considering these limitations, our goal is to develop a novel representation that is more capable of describing stripe nonuniformity in IR images, and then use it to design an effective stripe nonuniformity correction architecture. To this end, we explore the stripe observation model and its corresponding spectral characteristics that enable the method to efficiently extract stripe nonuniformity from image structures even in the heavy noise case. Our idea is as follows.

First, we introduce a spectral representation of stripe nonuniformity noise. It accurately depicts differential distributions of the noise and image structures in frequency domain, which is capable of discriminating these two parts in an image.

Second, we propose a two-stage filtering based stripe nonuniformity correction method for single IR image. Extensive experiments and evaluations demonstrate that our method outperforms state-of-the-art methods on both simulated images and raw IR images. Particularly for some heavy stripe nonuniformity images, our method achieves considerably good correction results.

The rest of this paper is organized as follows. In Section 2, related work on various NUC methods is reviewed. In Section 3, spectral representation of stripes as a priori foundation is presented. Section 4 describes the proposed two-staged filtering approach and more implementation details are given in Section 5. Section 6 provides experimental results to verify the performance of the proposed method. Finally, conclusions are drawn in Section 7.

## 2. Related Work

We review relevant recent work on the FPN removal including conventional NUC methods and specific correction methods for single image stripe nonuniformity.

**Conventional Nonuniformity Correction**. There are two main categories in conventional NUC: the calibration based methods and the scene based methods. The first class is simple and practical, for example the two-point calibration method [5]. It uses a surface source blackbody as the reference to calibrate detectors response by measuring the radiations at two distinct and known temperatures. More temperature segmentations can produce more accurate correction results [6]. In the second class, several typical strategies have been used such as the constant statistics methods [1,8], the filtering estimation methods [9,10], the image registration methods [11,12] and so on. Generally, these scene-based NUC approaches seek to make a reasonable estimate for model parameters, based on the FPN’s time-invariance. They are more applicable to general unstructured FPN cases by exploiting a sequence of IR images.

**Single Image Based Stripe Nonuniformity Correction**. Compared to multi-frame based NUC methods, stripe nonuniformity can be individually corrected only via a single image due to its distinctive appearance. There has been a lot of relevant research to tackle the problem, which can be grouped into grayscale-statistics-based [13,16,17], spatial-filtering-based [14,18,19] and constrained-optimization-based [15,20] methods. The first fashion considers the column-wise gray statistics of the image and assumes that the histograms of adjacent columns should be essentially the same. So the new histogram of each column can be modified by correlating histograms of neighboring columns. The second one applies appropriate spatial filters to smooth stripe nonuniformity, whose performance declines significantly in the strong noise case. The last fashion models the problem as energy function minimization. It assumes that the vertical gradient of the image should be sustained while the horizontal gradient seriously interfered by stripe nonuniformity should be as smooth as possible. More recently, with the advances of convolutional neural networks (CNNs) in low-level image understanding, Kuang et al. [21] firstly proposed a deep CNN model to correct stripe nonuniformity in a single IR image. Following this work, a residual deep network added by a column FPN simulation module was also proposed in [22], which achieves an accurate estimate of stripe nonuniformity.

More related with our work is the spectral filtering based methods and two-stage spatial filtering methods. Munch et al. [23] proposed a combined wavelet-Fourier filtering method for stripe artifact removal, where stripe noise is first separated into vertical high-frequency subbands by wavelet transform and then the Fourier coefficients of these vertical subbands are filtered with a Gaussian damping filter. Its success demonstrates the effectiveness of spectrum characteristics of stripes as an image prior in the destriping task. Unfortunately, as the literature [20] points out, the method is likely to produce smearing artifacts along the vertical edges. The underlying reason lies in the aggressive nature of the designed spectral filter, making low-frequency components that represent the grayscale information become erased with noise frequency reduction. In addition, Cao et al. [14] presented a two-stage guided filtering stripe noise removal method for a single IR image, where 1-D row and column guided filters are adopted in two stages, respectively. Due to only filtering in the spatial domain, the method inevitably takes away some detailed information overlapped with stripe noise when it is performed.

## 3. Prior Foundation

We briefly discuss the spectral characteristic of stripe nonuniformity noise, and then generalize it to design the observation model both in spatial and frequency domains.

### 3.1. Spectral Analysis of Stripe Noise

**Figure 1:**
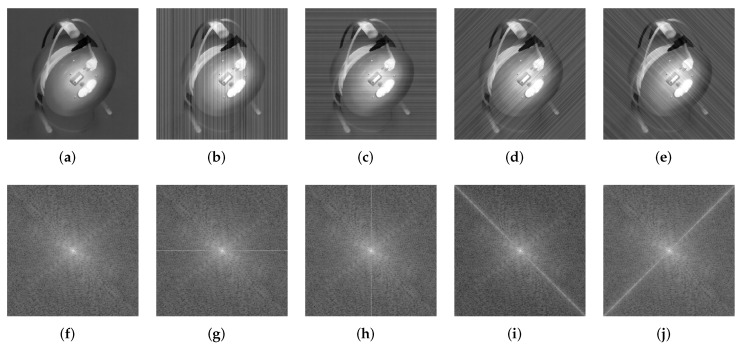
Spectral behavior of stripe noise with different directions. (**a**) Clean image; (**b**–**e**) Noisy images with vertical, horizontal, right diagonal and left diagonal stripes, respectively; (**f**–**j**) Amplitude spectrums corresponding to (**a**–**e**).

It has been proved that the Fourier amplitude spectrum of image components will be a highlighted spectrum line and orthogonal to themselves compared with others without any regularity, only when they have fixed orientations, such as vertical and horizontal. This intuition attributes to energy focusing of Fourier amplitude spectrum of image components. Based on this point, we can easily find that the Fourier amplitude spectrum of stripe noise follows this objective law since it has a fixed direction. To further reveal this objective law, we give an instance as shown in Figure 1. The top row gives the clean image and its corresponding noisy versions with different directional stripe noise, and the bottom row shows the Fourier amplitude spectrum corresponding to top row images. From Figure 1f–j, it can be easily seen that a bright line regularly adheres to the original image spectrum, which is orthogonal to the corresponding stripes. To illustrate this phenomenon, a theoretical analysis is given as follows.

Assume an ideal vertical stripe *V* in the image should be a constant *c* at any pixel position, which can be expressed as

(1) $$V\left(x,y\right)=\left\{\begin{array}{cc} c,\; & \mathrm{if}\;\left(x,y\right)\in \Omega^{W\times H}\\ 0,\; & \mathrm{otherwise} \end{array}\right.$$

where (x,y) denotes the image pixel, $\Omega^{W\times H}$ is the region of *V*, and *W* and *H* are its horizontal width and vertical height, respectively. Since vertical stripe is very thin, *W* obeys W≪H. The discrete Fourier spectrum of *V* is expressed as

(2) $$F\left(u,v\right)=cWH\frac{\sin(\pi uW)}{\pi uW}\frac{\sin(\pi vH)}{\pi vH}e^{-j\pi (uW+vH)}$$

where *u* and *v* are horizontal and vertical frequency coordinates, both $\frac{\sin(\pi uW)}{\pi uW}\frac{\sin(\pi vH)}{\pi vH}$ is a representation of 2-D Sinc function. The corresponding amplitude spectrum is

(3) $$\left|F\left(u,v\right)|=cWH\right|\frac{\sin(\pi uW)}{\pi uW}\left|\right|\frac{\sin(\pi vH)}{\pi vH}|$$

**Figure 2:**
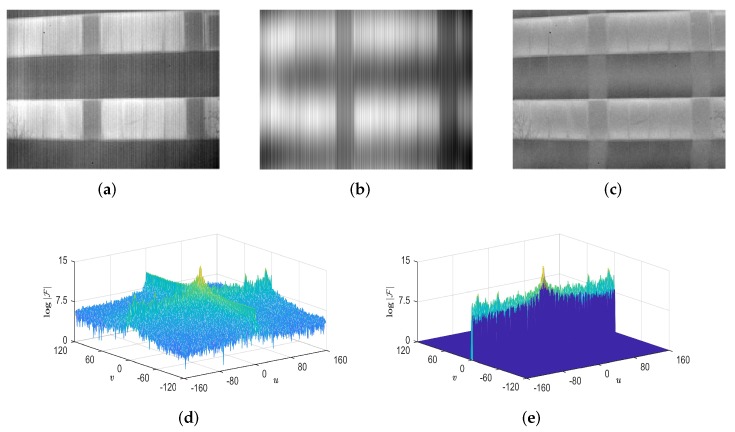
Example of image representation using the stripe frequency band (SFB). (**a**) Real IR image with stripe nonuniformity noise; (**b**) Noise component reconstructed by the SFB; (**c**) Structures in the remaining component; (**d**) Amplitude spectrum of (**a**); (**e**) SFB separated from (**d**).

According to the absolute Sinc function, |F| will shrink to *u* axis since 1/W≫1/H and show a comb-like impulse spectrum for the ideal stripe *V*. That is to say, |F| extends only in v=0, perpendicular to the extending direction of *V* in the image domain. The conclusion is also valid to striping patterns in other directions. Further, for stripe nonuniformity noise in the real IR image, we found that its frequencies are located in a narrow horizontal band that centres on the whole *u* axis. The band is referred to as stripe frequency band (SFB). Figure 2 shows an example of image representation using the SFB. We can see that the image component reconstructed by the SFB includes almost all stripe noise, while the structure information of the image is intactly reserved in the remaining component. This reveals an effective spectral prior for stripe nonuniformity noise. However, it should be noted that the noise component holds significant grayscale information as well because low frequencies of the image are located in the central part of the SFB.

### 3.2. Observation Model

A typical observation model in spatial domain for single IR image with the FPN can be formulated as

(4) D(x,y)=m(x,y)I(x,y)+a(x,y)

where I(x,y) and D(x,y) are the ideal image and its degraded observation at pixel (x,y), respectively. The FPN is generally modeled by a multiplicative component m(x,y) and an additive component a(x,y) based on the response characteristic of IR detectors. m(x,y) is generated from the difference between the response gains of detectors, while a(x,y) is caused by detectors’ biases variation.

For the striping structured FPN, the aforementioned formulation can often be simplified as an additive process because of its particularity. On the one hand, stripe nonuniformity arises from column-parallel integration readout circuits, where one amplifier is shared in each column [15,22]. On the other hand, the additive model has been proven effective to remove stripe noise solely in many applications [24,25]. So we model the IR image observation with stripe nonuniformity as

(5) D(x,y)=I(x,y)+S(x,y)

where S(x,y) denotes stripe nonuniformity noise. Due to the Fourier transform being linear, we have a corresponding spectral observation model as follows.

(6) D(u,v)=I(u,v)+S(u,v)

where D(u,v), I(u,v) and S(u,v) are the corresponding image representation in frequency domain.

The additive model expressed in Equations (5) and (6) suggests that stripe nonuniformity appearing on the observed image can be corrected in spatial domain and frequency domain as long as it is represented well. Inspired by this point, this paper attempts to solve the task for single IR image in a staged filtering way. The details of the proposed method will be elaborated in Section 4.

## 4. Proposed Method

**Figure 3:**
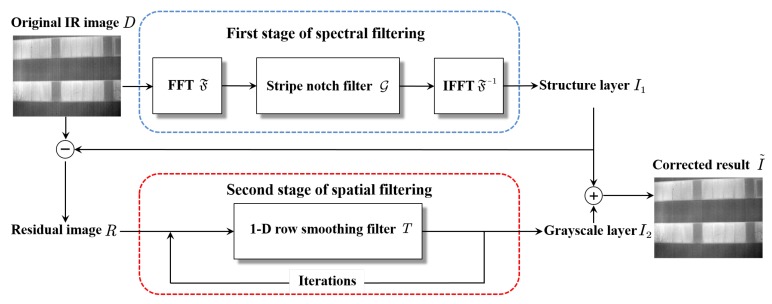
Framework of the proposed method.

Our goal is to take a single stripe nonuniformity IR image and output a corrected image. Figure 3 shows the framework of the proposed method. Its key idea is combining spectral filtering and spatial filtering to progressively eliminate stripe nonuniformity without blurring any fine image details. Firstly, the original IR image *D* in frequency domain is filtered by a stripe notch filter G that takes the SFB as its rejection region. The filtered result is called the structure layer $I_{1}$ that contains the complete image structure but lacks grayscale information. Then the residual image *R* between *D* and $I_{1}$ is used to estimate the grayscale layer $I_{2}$. Because of excessive stripe noise appearing in *R*, the operation of 1-D row smooth filtering with multiple iterations is adopted. The corrected result $\tilde{I}$ is easily obtained by adding the two image layers. The details of the proposed method are presented in the following subsections.

### 4.1. Stage 1: Spectral Filtering with Stripe Notch Filter

Notch filter is one of the most selective frequency filters and capable of rejecting or passing frequencies in predefined regions [26]. Inspired by this theory, we first define a stripe notch filter (SNF) with spectral prior knowledge that takes the SFB as its rejection region. Thus it can maximally separate the image structure from stripe nonuniformity. Based on this point, the basic flow of the spectral filtering stage consists of three steps.

**Step 1:** Transform the original IR image into the frequency domain via 2-D fast Fourier transformation (FFT).

Given a degraded image *D* with size of M×N, its amplitude spectrum A and phase spectrum P can be obtained by

(7) A=R[F(D)]

(8) P=P[F(D)]

where F, R and P denote the operation of 2-D FFT, amplitude and phase calculation, respectively. It should be noted that the phase spectrum P is indispensable to the subsequent inversion, which contains the location information of image structure [27].

**Step 2:** Construct the SNF whose rejection region is fixed as the SFB.

The SFB is loosely considered as the boundary between stripe nonuniformity noise and image structure in frequency domain. Rather than the identification of the noise frequencies with great precision, such a semi-accurate design greatly reduces the difficulty and complexity of the notch filter in construction. In addition, it is efficient and robust to various stripe nonuniformity images.

For convenience, we use $G\in R^{M\times N}$ to represent SNF, it can be defined by a simple binary function

(9) $$G\left(u,v\right)=\left\{\begin{array}{cc} 0,\; & \mathrm{if}\left(u,v\right)\in \Theta^{K\times N}\\ 1,\; & \mathrm{otherwise} \end{array}\right.$$

where $\Theta^{K\times N}$ denotes the SFB with size of K×N. The element of 0 means that the frequency is rejected, while 1 indicates that the frequency is allowed to pass. In this definition, there is only one parameter *K*, representing the number of rows within the SFB. It determines the stop scope of the SNF and is set to 2 in this study.

**Step 3:** Mask the amplitude spectrum with the well-defined SNF, and then perform the inverse 2-D FFT on the new Fourier spectrum.

Using the defined SNF G, we can obtain the structure layer $I_{1}$ by

(10) $$A^{{}^{\prime }}=G⊙A$$

(11) $$I_{1}=F^{-1}\left(A^{{}^{\prime }}e^{jP}\right)$$

where $A^{{}^{\prime }}$ is the filtered version of A, and the operators of ⊙ and $F^{-1}$ denote the element-wise multiplication and the inverse 2-D FFT, respectively.

### 4.2. Stage 2: Spatial Filtering with 1-D Row Smoothing Filter

After the first stage, the grayscale information is deposited into the residual image *R* with stripe nonuniformity, since low-frequency parts of the image are also rejected by G. To further separate these two parts, we employ a 1-D row smoothing filter to process *R* via a few iterations. After finishing the iterative refinement, the grayscale layer $I_{2}$ can be easily estimated from *R* without stripe nonuniformity.

The spatial filtering of the residual image *R* can be expressed as

(12) $$I_{2}^{\left(i+1\right)}=I_{2}^{\left(i\right)}⋆T$$

where ⋆ denotes the spatial filtering operation and *i* is the *i*-th iteration. In the implementation, let $I_{2}^{\left(0\right)}=R$ for initialization and *T* can be selected from existing spatial filters. In addition, the total number of iterations is symbolized $i_{\max}$, which is an experiential parameter to be set in the procedure. The details will be described in Section 5.1.

Once the grayscale layer is obtained, the final corrected result $\tilde{I}$ can be achieved by

(13) $$\tilde{I}=I_{1}+I_{2}$$

## 5. Implementation Details

In this section, we present implementation details of the proposed method. First, some notes on filters used in the two stages are elaborated. Then the entire procedure is given as a quick reference.

### 5.1. Notes on Filters

For the M×N image (*M* and *N* are even by default), the horizontal center of its frequency spectrum after the origin shifting operation is located at row (M/2+1). Thus in G’s construction, Θ is centered on the (M/2+1)-th row and has *K* rows. That is, K=2 means that the elements in rows (M/2+1) and M/2 (or (M/2+2)) are filled with 0 and by analogy, the three rows are set as Θ if K=3. Note that *K* should be assigned small integer values because of the narrow SFB.

In the realization of spatial filtering, we adopt a combination of two traditional smooth filters, mean filter $T_{1}$ and gaussian filter $T_{2}$, to play the role of *T*. Both filters are alternately used in the iterative refinement. Rather than the use of a single filter, such a combination of two varying filters is more efficient for the estimation of $I_{2}$. The default is the same size of $T_{1}$ and $T_{2}$ as 1×5. The standard deviation of the Gaussian kernel in $T_{2}$ is set to 1.2. Besides, the total number of filtering iterations $i_{\max}$ is also an important parameter in the second stage. Its value should be flexibly adjusted with the noise intensity for achieving better performance. An appropriate range is suggested to be 2∼50 and we found that it would become less significant for improving the corrected result when the value is larger than 50. A detailed discussion about this will be given in a later section.

### 5.2. Procedure

The entire procedure of the proposed method is summarized in Algorithm 1.

**Algorithm 1.** Two-stage filtering method for single IR image stripe nonuniformity correction**Input:** The original IR image *D*. **Stage 1: Spectral filtering with stripe notch filter G.** **Parameter:** The width *K* of G’s rejection region. **Forward transformation.** Apply 2-D FFT on *D* to obtain the corresponding Fourier spectrum, and then calculate the amplitude A and the phase P separately. **Filtration.** Correlate A with G to get the filtered amplitude $A^{{}^{\prime }}$, and calculate the modified Fourier spectrum $A^{{}^{\prime }}e^{jP}$. ** Backward transformation.** Apply inverse 2-D FFT, and obtain the structure layer $I_{1}$. **Stage 2: Spatial filtering with 1-D row smoothing filter**
*T***.** (Use mean filter $T_{1}$ and gaussian filter $T_{2}$) **Parameter:** The total number of filtering iterations $i_{\max}$. **Initialization:** Let the residual image $R=D-I_{1}$ be the initial grayscale layer $I_{2}^{\left(0\right)}$. **for**
i=1 to $i_{\max}$ do  Perform spatial filtering alternately on $I_{2}^{\left(i\right)}$ by $T_{1}$ and $T_{2}$ to refine $I_{2}$. **end for** **Output:** The final corrected result $\tilde{I}=I_{1}+I_{2}$.

## 6. Experiments

In this section, the performance of the proposed method is tested on simulated images and raw IR images. We present the quantitative evaluation, qualitative comparison, run time, and parameters analysis. Here, we compare with four state-of-the-art stripe nonuniformity correction algorithms, including the combination of wavelet transform and spectral filtering (WTSF) [23], MIRE [13], guided filtering (GIF) [14] and CNN [21]. In addition, all the free parameters of these compared methods are set to default values following with the corresponding references.

### 6.1. Data Sets

**Figure 4:**
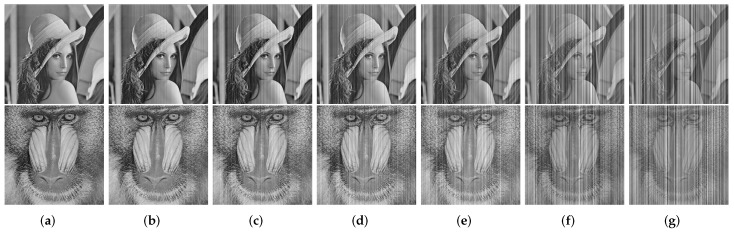
Two sets of simulated noisy images for testing. (**a**) Clean images Lena and Mandrill; (**b**–**g**) Noisy images Lena and Mandrill with stripe noise of σ=0.02,0.04,0.08,0.16,0.32, respectively.

**Figure 5:**
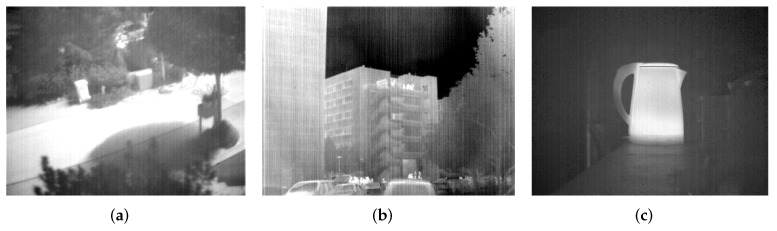
Three raw IR images for testing. (**a**) Street from ASL dataset [28]; (**b**) Building from Tendero’s dataset [13]; (**c**) Kettle from our dataset.

In this work, we carry out extensive experiments on two kinds of images. The first one is gray test images Lena and Mandrill, which are artificially added with simulated stripe noise as the corresponding nonuniformity image observations. The noise addition is followed by the model (5) and assumed as 1-D random Gaussian process with zero mean and standard deviation σ. We generate five stripe noise images with different σ values, as shown in Figure 4. The other one is three raw IR images as shown in Figure 5. The Street and Building images come from ASL dataset [28] and Tendero’s dataset [13], respectively. Kettle is from our dataset. These raw images are recorded by different IR sensors and slightly or heavily corrupted by stripe nonuniformity. Detailed description about the test data is listed in Table 1.

### 6.2. Test on Simulated Images

In the simulation experiment, the destriping performance is evaluated by two common full-reference image quality metrics, the peak signal to noise ratio (PSNR) and structure similarity (SSIM) index [29]. PSNR measures pixel errors between the filtered image and its referenced image. A higher PSNR value indicates a smaller image distortion. It is defined as

(14) $$\mathrm{PSNR}=10\log_{10}\frac{255^{2}}{\mathrm{MSE}}\;\;\left(\mathrm{dB}\right)$$

where MSE is the mean squared error between the filtered and referenced images. SSIM provides a perceptually relevant assessment for the filtered result, which is calculated within a sliding window by using

(15) $$\mathrm{SSIM}(m,n)=\frac{\left(2\mu_{m}\mu_{n}+C_{1}\right)\left(2\sigma_{mn}+C_{2}\right)}{\left(\mu_{m}^{2}+\mu_{n}^{2}+C_{1}\right)\left(\sigma_{m}^{2}+\sigma_{n}^{2}+C_{2}\right)}$$

where m and n are the image patches extracted by the sliding window from the filtered and referenced images, respectively. $\mu_{m}$, $\mu_{n}$, $\sigma_{m}$, $\sigma_{n}$, and $\sigma_{mn}$ are the mean, standard deviation and cross-correlation of m and n, respectively. $C_{1}$ and $C_{2}$ are very small constants that avoid zero denominator error. In actual measurements, the mean SSIM of two images is computed as the final SSIM score. It is a decimal value between 0 and 1. The closer the SSIM value is to 1, the better structure-preserving effect the filtered image has.

Table 2 and Table 3 show the values of PSNR and SSIM for different methods, respectively. They are obtained by averaging 10 independent realizations of the noise process. Compared with the four methods, it can be easily found that the proposed method achieves the highest scores in terms of both PSNR and SSIM. This demonstrates that our proposed method outperforms the other methods.

**Figure 6:**
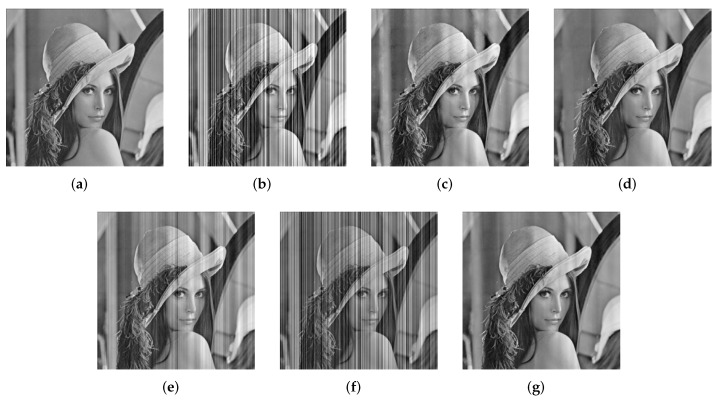
Filtered results for Lena corrupted by stripe niose with σ=0.16. (**a**) Clean; (**b**) Noisy; (**c**) WTSF; (**d**) MIRE; (**e**) GIF; (**f**) CNN; (**g**) Ours.

To intuitively illustrate that our proposed method is capable of getting superior performance, an example of stripe noise removal is given as shown in Figure 6. One can clearly see that the results of GIF and CNN cannot be accepted, when the clean image is corrupted by stripe noise with σ=0.16. There are still apparent striping atrifacts in these two results. In contrast, the results obtained by WTSF and MIRE seem somewhat better. However, they produce unnatural grayscale degradation and distortion in local regions. The proposed method achieves the best visual effect, where all the stripe noise is reduced but some detailed information is blurred.

On the whole, the proposed method in eliminating stripe noise achieves remarkable results for simulated images both in visual effect and objective metrics. In the following section, we will focus on raw IR images with stripe nonuniformity.

### 6.3. Test on Raw IR Images

**Figure 7:**
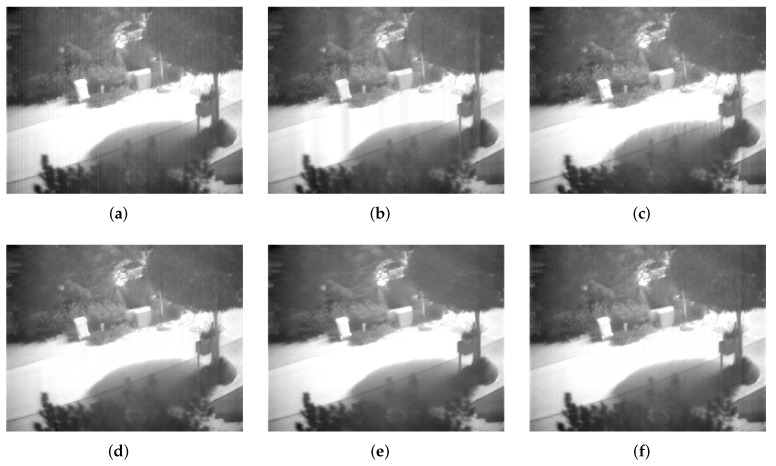
Stripe nonuniformity correction results for Street. (**a**) Raw; (**b**) WTSF; (**c**) MIRE; (**d**) GIF; (**e**) CNN; (**f**) Ours.

**Figure 8:**
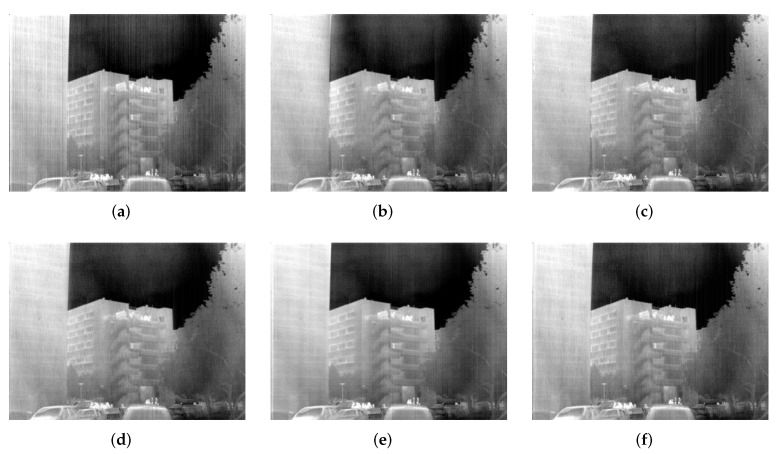
Stripe nonuniformity correction results for Building. (**a**) Raw; (**b**) WTSF; (**c**) MIRE; (**d**) GIF; (**e**) CNN; (**f**) Ours.

**Figure 9:**
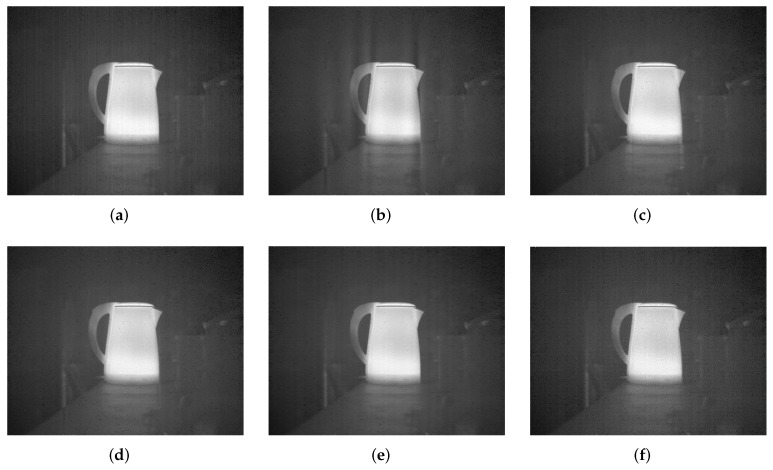
Stripe nonuniformity correction results for Kettle. (**a**) Raw; (**b**) WTSF; (**c**) MIRE; (**d**) GIF; (**e**) CNN; (**f**) Ours.

Corrected results for Street, Building and Kettle are shown in Figure 7, Figure 8 and Figure 9, respectively. It can be observed that WTSF and MIRE produce some conspicuous column-wise artifacts. In the nonuniformity noise-friendly cases (such as Street and Kettle), the results obtained by CNN are attractive and have a soft visual effect. However, its correction ability becomes less effective when dealing with heavy nonuniformity (see the case of Building), as verified in the simulated experiment. For these raw IR images, GIF and the proposed method achieve similar and good stripe nonuniformity correction results visually.

**Figure 10:**
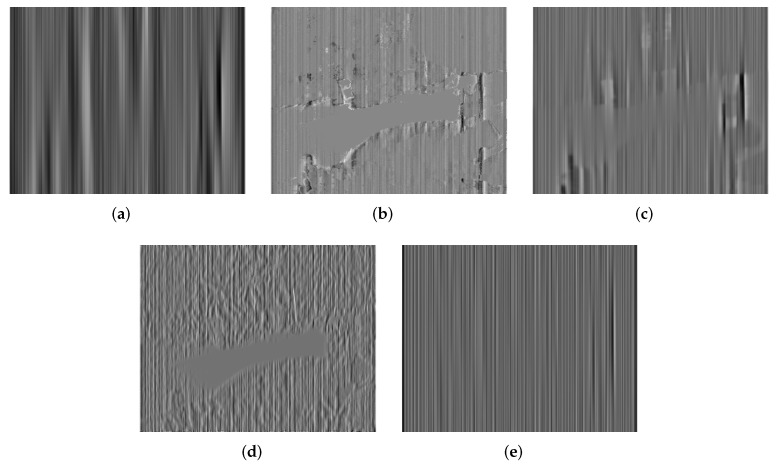
NUN results for Street. (**a**) WTSF; (**b**) MIRE; (**c**) GIF; (**d**) CNN; (**e**) Ours.

**Figure 11:**
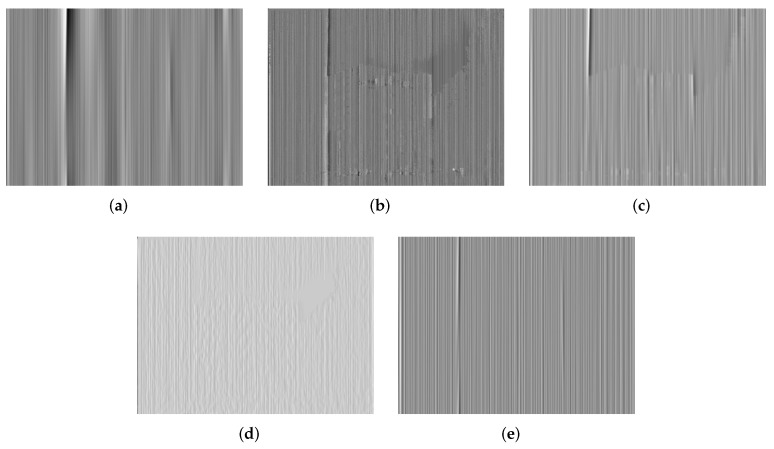
NUN results for Building. (**a**) WTSF; (**b**) MIRE; (**c**) GIF; (**d**) CNN; (**e**) Ours.

**Figure 12:**
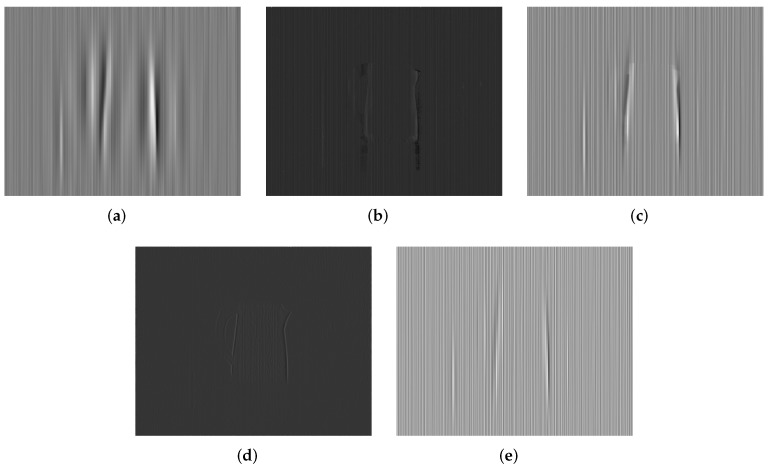
NUN results for Kettle. (**a**) WTSF; (**b**) MIRE; (**c**) GIF; (**d**) CNN; (**e**) Ours.

Additionally, we make use of the nonuniformity noise (NUN) image that is a difference result between the original and corrected images [14,15] to illustrate those imperceptible discrepancies in the visual results. The ideal NUN image produced by a good correction method should be a pure stripe noise pattern. On the contrary, for an inferior correction method, there is apparent structure information in the NUN image, such as edges or textures. The NUN results for three raw IR images are shown in Figure 10, Figure 11 and Figure 12, respectively. It is found that the NUN image obtained by MIRE includes distinct structure information, followed by GIF. WTSF greatly affects the intensity change around strong vertical edges. From the NUN results in Figure 12d and Figure 14d, CNN can smooth the whole image but reduce a few image details, such as the textures of the tree crown and the outline of the kettle. By contrast, the NUN image acquired by the proposed method includes minimal structure information. From this point of view, the proposed method performs much better than the other methods in detail-preserving.

Besides visual comparisons, we also apply two quantitative indicators to evaluate the correction performance of the proposed method objectively. The roughness index ρ [16,22] is commonly used to measure the nonuniformity in IR images. It is defined as

(16) $$\rho \left(G\right)=\frac{{∥f_{1}⊗G∥}_{1}+{∥f_{2}⊗G∥}_{1}}{{∥G∥}_{1}}$$

where *G* is the IR image to be measured, $f_{1}=\left[1,-1\right]$ and $f_{2}=f_{1}^{T}$ represent the 1-D horizontal and vertical differencing filters, respectively. ${∥\cdot ∥}_{1}$ is the $L_{1}$ norm. By definition, ρ measures the nonuniformity substantially through calculating the gradient of *G*. So a smooth *G* tends to have a small ρ value, namely having low nonuniformity. Unfortunately, using such a metric has some limitations in terms of image quality evaluation, such as when the evaluated image is over-smoothing. For this reason, here we adopt the effective roughness index $\tilde{\rho }$ [4,30] instead of ρ to measure the nonuniformity, which is defined as

(17) $$\tilde{\rho }\left(G\right)=\rho \left(h⊗G\right)$$

where *h* is a high-pass filter as a preprocessing step and the $L_{2}$ norm is used in place of the $L_{1}$ norm for modification. Compared with ρ, $\tilde{\rho }$ estimates the nonuniformity after removing low-frequency components of the image. A smaller $\tilde{\rho }$ value indicates lower nonuniformity of the image. On the other hand, in order to evaluate detail-preserving ability of correction algorithms, we define the average vertical gradient error (AVGE) whose terms are given by

(18) $$\mathrm{AVGE}=\frac{1}{P}\sum_{p=1}^{P}\left|\right|\nabla_{y}\Gamma \left(Q_{p}\right)\left|-\right|\nabla_{y}Q_{p}\left|\right|$$

where $Q_{p}$ is the noisy image with pixel *p*, *P* is the number of pixels, Γ(·) is the destriping method, and $\nabla_{y}$ and |·| denote the vertical gradient operator and the absolute value operator, respectively. AVGE represents the variation of the vertical image gradient before and after correction. The principle behind it is based on the understanding that the vertical gradient should remain unchanged while the column nonuniformity is reduced. So the AVGE value closer to zero indicates that Γ is better at preserving image details.

The $\tilde{\rho }$ and AVGE results for raw IR images are reported in Table 4. The best results are emphasized in boldface. We can easily see that CNN and the proposed method yield smaller $\tilde{\rho }$ values and AVGE values. However, it should be pointed out that, for Building, the lowest AVGE value obtained by CNN may not be a positive result because CNN actually fails in this case. Overall, the quantitative evaluation results reveal the advantage of the proposed method in stripe nonuniformity correction and detail preservation.

Though testing on these raw IR images from different thermal imaging sensors, we demonstrate the effectiveness of our proposed approach in most real cases. Combining the qualitative and quantitative comparisons, we can conclude that the proposed method achieves the state-of-the-art stripe nonuniformity correction performance for raw IR images.

### 6.4. Time Consumption

We also consider time consumption of different methods to examine their computation complexity. In our experiments, all the test methods are implemented in Matlab R2017a on a common PC with an Intel Core i5 CPU (3.40 GHz) and 8 GB RAM. We make the statistics of the algorithm computing time on simulated and raw IR images with different resolutions. The average results are listed in Table 5. It can be seen that the proposed method requires a minimum of computing time in working on the same image, compared to the other methods. With the resolution enhancing of the input image, time consumption of the proposed method can stabilize in a low range. This implies the potential of our method in the real-time processing.

### 6.5. Parameter Analysis

**Figure 13:**
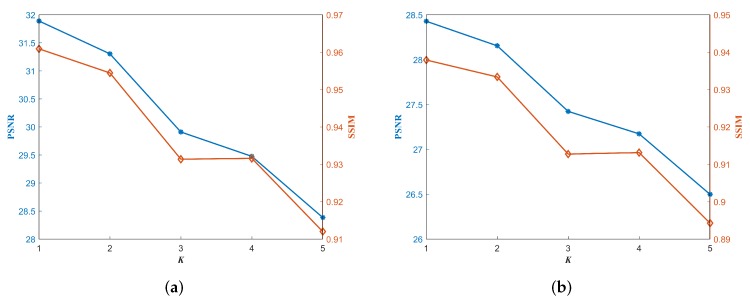
Two sets of PSNR (dB) and SSIM results for Lena with different *K* values respectively in the cases of adding stripe noise of σ=0.08 and 0.16. (**a**) PSNR and SSIM when σ=0.08; (**b**) PSNR and SSIM when σ=0.16.

In this section, we analyze the influences of two main parameters in the proposed approach, *K* and $i_{\max}$. We first fix $i_{\max}=10$ and other default parameters to just consider *K* in a small range of 1∼5. In Figure 13, two sets of PSNR and SSIM results for Lena with different *K* values are reported respectively in the cases of adding stripe noise of σ=0.08 and 0.16. It can be observed that both groups are similar and the quality of the denoised image is decreased with the increase of *K* value. In detail, the losses of PSNR and SSIM are minor when *K* increases from 1 to 2, while the losses become serious when *K* increases from 2 to 3. Thus, it is reasonable to set *K* to be 2 in this paper.

**Figure 14:**
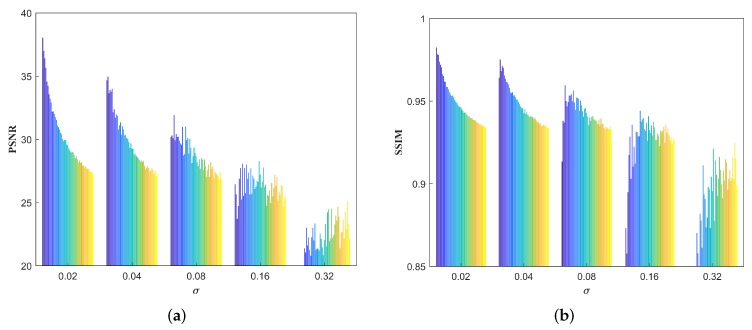
PSNR (dB) and SSIM results for Lena with different $i_{\max}$ values in the five stripe noise cases. (**a**) PSNR; (**b**) SSIM.

We also take $i_{\max}$ as the only variable to investigate the selection of its value within a range of 2∼50. Figure 14 shows PSNR and SSIM results for Lena with different $i_{\max}$ values in the five stripe noise cases. For each case, the histograms from blue to yellow represent changes in PSNR and SSIM values with the increase of $i_{\max}$ from 2 to 50. It can be easily found that PSNR and SSIM has higher scores when the value of $i_{\max}$ is smaller in the cases of σ=0.02,0.04,0.08, while in the cases of σ=0.16,0.32 higher PSNR and SSIM scores are obtained when setting a larger $i_{\max}$ value. The reason for this is that in most cases (σ=0.02,0.04,0.08) the noise is relatively slight or moderate so that low filtering iterations tend to maintain the image grayscale while suppressing the noise. On the contrary, the noise becomes the main component and severely corrodes image pixels when it is very heavy like the case of σ=0.16 or even 0.32. Thus, enough filtering iterations are more effective.

**Figure 15:**
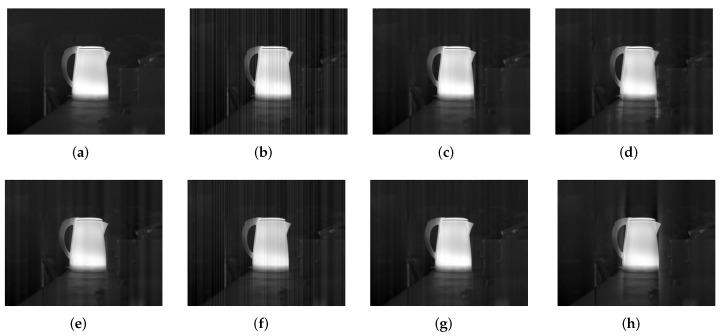
Filtered results for Kettle corrupted by stripe niose with σ=0.04. (**a**) Clean; (**b**) Noisy; (**c**) WTSF; (**d**) MIRE; (**e**) GIF; (**f**) CNN; (**g**) Ours with $i_{\max}=2$; (**h**) Ours with $i_{\max}=40$.

**Figure 16:**
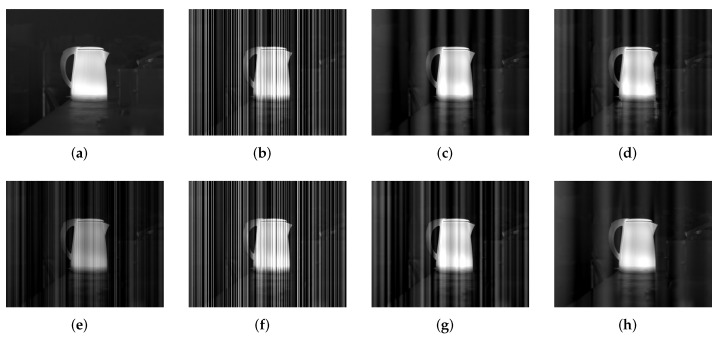
Filtered results for Kettle corrupted by stripe niose with σ=0.32. (**a**) Clean; (**b**) Noisy; (**c**) WTSF; (**d**) MIRE; (**e**) GIF; (**f**) CNN; (**g**) Ours with $i_{\max}=2$; (**h**) Ours with $i_{\max}=40$.

For a further comparison, Figure 15 and Figure 16 show IR image examples for Kettle (an ideal output from the camera) corrupted by stripe noise with σ=0.04 and σ=0.32, respectively, when $i_{\max}$ is set to be 2 and 40 in our method. The corresponding PSNR and SSIM results are listed in Table 6. From these results, we can also conclude that a small $i_{\max}$ value is favourable when the noise is slight, while $i_{\max}$ should be set to be a large value in the strong noise case. When $i_{\max}$ has a larger value in the case of σ=0.04, stripe noise is suppressed further but some undesirable artifacts also emerge, especially near the vertical edge that is similar to stripes. This is an over-suppression effect. In contrast, as we mentioned before, the noise in the case of σ=0.32 becomes the relatively main component in the image, where a small $i_{\max}$ value fails to obtain an acceptable result.

However, practically speaking, there are certain differences between the simulated noise and the real nonuniformity noise in appearance. The real noise intensity and fluctuation are relatively mild in raw IR images. On the one hand, this can be attributed to the current advanced IR detector technology. On the other hand, IR image that is a reflection of temperature difference in distribution always appears globally smooth, including its noise pattern. Because thermal equilibrium occurs in the recorded scene. Given this, a small $i_{\max}$ value is suggested in filtering on raw IR images in our approach.

### 6.6. More Discussion

**Figure 17:**
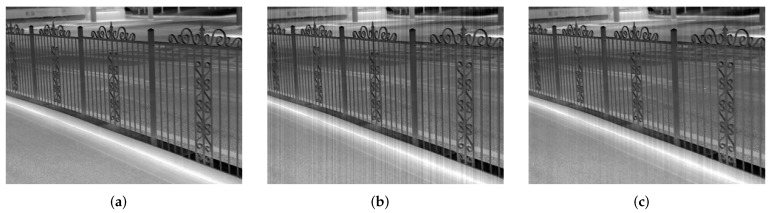
Filtered result for Fence (**a**) Clean; (**b**) Noisy; (**c**) Corrected.

In this section, we discuss destriping in thermal images for some frequent objects that have a similar appearance with stripe noise in the image, such as radiator and regular fence or trees. Figure 17 shows an example for Fence taken from [31], in which stripe noise with σ=0.04 is added. The PSNR and SSIM results are also given in Table 7. From the results, it can be illustrated that the proposed method still has a good destriping performance on the image having a similar striping structure. In essence, stripe noise has its own manifestation features to distinguish itself from the similar patterns. (1) The noise entangles itself in each pixel and its intensity in the same column can be considered approximately identical. (2) These stripes vary visibly between columns and extend throughout the whole image. (3) There is nothing for the noise in other features such as shape, compared with object patterns similar to it. So the noise can be effectively controlled in these images. However, we also notice that some artifacts are generated inevitably in the corrected result because of the striping similarity. In this point, our approach should be improved further.

## 7. Conclusions

In this work, we propose a new stripe nonuniformity correction method for single IR image. Our model progressively eliminates stripe nonuniformity noise in a coarse-to-fine manner with two-stage filtering. By analyzing the significant spectral difference between stripe noise and image structures, we build on this characteristic to achieve improved performance in stripe nonuniformity correction and detail preserving. The effectiveness of the proposed method is validated by testing on simulated images and raw IR images.

Future work will focus on exploring the spectral distribution of stripe noise further. Developing an adaptive nonuniformity noise frequency detector is an appealing topic. In addition, more prior knowledge of IR image and more object scenes will be taken into account for stripe nonuniformity correction.

## Figures and Tables

**Table 1:** The details of the test data.

Test Data	Source	Size	Sensor	Description
Simulated images				
Lena Mandrill	——	256×256	——	Widely used gray images, added with different levels of stripe noise.
Raw IR images				
Streat	ASL dataset	324×256	Handheld FLIR Tau 320 camera	Outdoor image, rich nature scene, small details and obvious stripe nonuniformity.
Building	Tendero’s dataset	384×288	Thales Minie-D camera	Outdoor image, regular lines and edges and obvious stripe nonuniformity.
Kettle	Our dataset	640×512	LUSTER TB-M640-CL camera	Indoor image, simple object scene, legible outline, and slight stripe nonuniformity.

**Table 2:** Peak signal to noise ratio (PSNR) (dB) results for Lena and Mandrill.

σ	Lena							Mandrill					
	Noisy	WTSF	MIRE	GIF	CNN	Ours		Noisy	WTSF	MIRE	GIF	CNN	Ours
0.02	33.97	31.50	24.82	33.63	36.90	**37.66**		34.06	35.27	32.52	36.39	35.33	**38.21**
0.04	28.02	31.02	24.78	32.63	31.27	**33.88**		28.02	34.25	32.26	34.54	30.19	**35.50**
0.08	22.06	29.52	24.62	29.73	23.73	**30.39**		22.15	32.14	31.34	30.99	23.54	**33.07**
0.16	15.99	26.25	23.79	24.13	16.61	**27.02**		15.95	27.21	28.95	24.16	16.49	**29.08**
0.32	10.27	21.16	21.80	15.92	10.68	**22.67**		10.08	21.50	24.90	15.59	10.49	**25.07**

**Table 3:** Structure similarity (SSIM) results for Lena and Mandrill.

σ	Lena							Mandrill					
	Noisy	WTSF	MIRE	GIF	CNN	Ours		Noisy	WSTF	MIRE	GIF	CNN	Ours
0.02	0.894	0.942	0.920	0.971	0.979	**0.982**		0.965	0.984	0.985	0.990	0.983	**0.993**
0.04	0.718	0.941	0.921	0.965	0.884	**0.969**		0.888	0.984	0.985	0.987	0.945	**0.991**
0.08	0.480	0.932	0.920	0.932	0.585	**0.953**		0.728	0.981	0.984	0.976	0.790	**0.988**
0.16	0.260	0.899	0.914	0.745	0.290	**0.932**		0.462	0.968	0.982	0.889	0.497	**0.984**
0.32	0.111	0.817	0.901	0.334	0.121	**0.911**		0.215	0.927	0.973	0.534	0.225	**0.976**

**Table 4:** $\tilde{\rho }$ and AVGE results for raw IR images.

	Street			Building			Kettle	
	$\tilde{\rho }$	AVGE		$\tilde{\rho }$	AVGE		$\tilde{\rho }$	AVGE
Raw	1.229	—		2.042	—		2.565	—
WTSF	1.101	0.031		1.270	0.036		1.796	0.005
MIRE	1.119	0.517		1.270	0.444		1.813	0.149
GIF	1.111	0.033		1.257	0.043		1.796	0.005
CNN	1.129	0.036		1.697	**0.015**		**1.749**	0.044
Ours	**1.073**	**0.010**		**1.253**	0.031		1.796	**0.001**

**Table 5:** Computing time (s) of different methods for simulated and raw IR images.

Image	Resolution	WTSF	MIRE	GIF	CNN	Ours
Lena/Mandrill	256×256	0.022	0.227	0.052	1.784	**0.017**
Street	324×256	0.039	0.306	0.074	2.125	**0.021**
Building	384×288	0.042	0.427	0.082	3.059	**0.024**
Kettle	640×512	0.088	1.685	0.195	14.390	**0.055**

**Table 6:** PSNR (dB) and SSIM results for Kettle corrupted by stripe noise with σ=0.04 and σ=0.32.

		Noisy	WTSF	MIRE	GIF	CNN	$i_{\max}=2$	$i_{\max}=40$
σ=0.04	PSNR	28.05	32.30	31.49	32.66	30.67	33.21	31.57
	SSIM	0.636	0.953	0.955	0.964	0.812	0.965	0.952
σ=0.32	PSNR	9.961	25.63	23.04	17.91	11.31	18.87	26.26
	SSIM	0.036	0.761	0.613	0.357	0.061	0.343	0.789

**Table 7:** PSNR (dB) and SSIM results for Fence.

	Noisy	Corrected
PSNR	29.82	33.66
SSIM	0.879	0.947
